# Dreaming of a New World Where Alzheimer’s Is a Treatable Disorder

**DOI:** 10.3389/fnagi.2019.00317

**Published:** 2019-11-15

**Authors:** Marcella Catania, Giorgio Giaccone, Mario Salmona, Fabrizio Tagliavini, Giuseppe Di Fede

**Affiliations:** ^1^Neurology V–Neuropathology Unit and Scientific Directorate, Fondazione IRCCS Istituto Neurologico Carlo Besta, Milan, Italy; ^2^Department of Molecular Biochemistry and Pharmacology, Istituto di Ricerche Farmacologiche Mario Negri IRCCS, Milan, Italy

**Keywords:** Alzheimer, amyloid-beta, multi-target, tau, amyloid cascade hypothesis, amyloid precursor protein, secretase, biometal ions

## Abstract

Alzheimer’s disease (AD) is the most common form of dementia. It’s a chronic and untreatable neurodegenerative disease with irreversible progression and has important social and economic implications in terms of direct medical and social care costs. Despite prolonged and expensive efforts employed by the scientific community over the last few decades, no effective treatments are still available for patients, and the development of disease-modifying drugs is now a really urgent need. The recent failure of clinical trials based on the immunotherapeutic approach against amyloid-β(Aβ) protein questioned the validity of the “amyloid cascade hypothesis” as the molecular machinery causing the disease. Indeed, most attempts to design effective treatments for AD have been based until now on molecular targets suggested to be implicated in AD pathogenesis by the amyloid cascade hypothesis. However, mounting evidence from scientific literature supports the view of AD as a multifactorial disease that results from the concomitant action of multiple molecular players. This view, together with the lack of success of the disease-modifying single-target approaches, strongly suggests that AD drug design needs to be shifted towards multi-targeted compounds or drug combinations acting synergistically on the main core features of disease pathogenesis. The discovery of drug candidates targeting multiple factors involved in AD would greatly improve drug development. So, it is reasonable that upcoming strategies for the design of preventive and/or therapeutic agents for AD point to a multi-pronged approach including more than one druggable target to definitely defeat the disease.

## Introduction

Alzheimer’s disease (AD) is a multifactorial neurodegenerative disorder characterized by progressive loss of neurons which may induce a decline in learning ability, loss of memory and impairment of other cognitive functions (Lane et al., 2018). It is the most common cause of dementia in the elderly (Goedert and Spillantini, 2006). Nearly 44 million people worldwide suffer from dementia and the number of patients is likely to reach 135 million in 2050; more than half of them will be AD cases (Prince et al., 2013). No new drugs have been approved for AD during the past 16 years and the available medications have very low impact on the disease course (Cacabelos, 2018), and even increase the costs for the care of AD patients by prolonging the length of a person’s “stay” in the Mild, Moderate, or Severe stage of disease (Cimler et al., 2019). For these reasons, AD is now recognized by the World Health Organization as a global public health priority.

Despite large gains in our understanding of AD pathogenesis, no disease-modifying treatments are still available for patients. Huge data reported in literature suggest that multiple factors such as amyloid-β (Aβ) assemblies, tau-protein aggregation and hyperphosphorylation, low levels of acetylcholine (ACh), mitochondrial dysfunction, oxidative stress, inflammation and dyshomeostasis of biometals might be actively involved in AD (Querfurth and LaFerla, 2010; Pfaender and Grabrucker, 2014; Stancu et al., 2014; Selkoe and Hardy, 2016; Wilkins and Swerdlow, 2016; Hardy, 2017). Over the years, the *amyloid cascade hypothesis* emerged as the dominant model of AD pathogenesis and is still driving the development of potential treatments targeting the main molecular players of AD (Walker et al., 2005; Chakraborty, 2017).

The recent failure of clinical trials based on monoclonal antibodies (mAbs) against Aβ imposes urgent dilemmas on the interpretation of mechanistic studies and leads us to a crucial crossroad among the hypotheses on AD pathogenesis and to a revision of strategies employed until now for the design of efficient treatments.

In this review article, we summarize the reasons for the ineffectiveness of the main experimental strategies targeting the molecular pathways suggested to be crucial for the disease, and highlight the urgent need for a radical change in the therapeutic approaches to AD. These approaches, in our opinion, should definitely point to a synergic strategy based on the concomitant use of multiple drugs or of a single multi-target compound to tackle the most relevant events in the molecular machinery that causes the onset of AD and its progression.

## On the Side of Amyloid Cascade Hypothesis or Beyond It?

AD pathophysiological hallmarks include Aβ plaques and neurofibrillary tangles (NFTs), which predominantly aggregate in the hippocampus and neocortex (Hyman et al., 2012). Aβ plaque deposition is associated with toxic soluble oligomers as well as eventual insoluble neuritic plaques (Hardy and Selkoe, 2002). For 25 years, the amyloid cascade hypothesis dominated the research on this disease (Hardy and Higgins, 1992; Selkoe and Hardy, 2016; Behl and Ziegler, 2017). It represented the almost exclusive source of the molecular targets for therapeutic strategies in AD and is supported by a long series of data reported in scientific literature during the last decades. The milestones in favor of this theory include genetic issues mainly provided by the discovery of pathogenic and protective mutations in the Amyloid Precursor Protein (APP; Selkoe, 1997; Di Fede et al., 2009; Jonsson et al., 2012; Hartley et al., 2015) and presenilin genes (Selkoe and Hardy, 2016), and the existence of polymorphisms in *ApoE* and other recently discovered genes modulating the risk of developing AD (Liao et al., 2017; Kunkle et al., 2019). Additional evidence comes from mechanistic, neuropathological and imaging studies indicating Aβ oligomers and hyperphosphorylated tau as key players in disease pathogenesis (Goedert and Spillantini, 2006; Wang et al., 2013; Selkoe and Hardy, 2016). However, the approaches against amyloid cascade players explored until now in clinical trials were unsatisfactory, despite promising results in the preclinical phases of their development (Pinheiro and Faustino, 2019).

### Immunotherapy Against Aβ

The strategy based on *passive immunotherapy* with anti-Aβ antibodies followed the perception that administering targeted antibodies is more effective than trying to induce their production *in vivo*. However, the first approaches based on passive immunotherapy against Aβ were carried out by using nonprotective mAbs against the Aβ N-terminal domain (Aβ1–15) that causes the release of cytotoxic Aβ oligomers which are immobilized as plaques, an event known as “dust raising effect” (Liu et al., 2015). Actually, most studies suggest that the immunogens needed to induce a protective immunity are the soluble Aβ oligomers, rather than either monomeric Aβ or plaques (Selkoe and Hardy, 2016) and that the critical epitopes are conformational rather than linear (Wisniewski and Drummond, 2016). However, clinical trials with antibodies like aducanumab, which differ from previous mAbs targeting *monomeric* Aβ, in that they recognize soluble cytotoxic Aβ protofibrils and oligomers provided negative results too. A possible explanation for this lack of efficacy may derive from the observation that almost all immunotherapeutic approaches against Aβ used the wrong Aβ-derived antigen, i.e., Aβ1–15, combined with proinflammatory adjuvants, e.g., QS-21 and CpG oligodeoxynucleotides, which elicited an undesirable pro-inflammatory immunity (Th1/Th17) rather than the required anti-inflammatory one (Th2; Marciani, 2015). In this view, the discrepancies between preclinical and clinical results may be explained by the fact that transgenic animals are more resilient than humans to the side effects of pro-inflammatory adjuvants. The aducanumab-based protocol is associated with a prevalent but not exclusive trigger of Th2-mediated immunity (Marciani, 2015). This could explain its better—but still unclear—results in comparison with other immunotherapeutic protocols.

An additional problem increasing the uncertainties on drug design for AD is Aβ pleomorphism. Increasing evidence suggests that different Aβ assemblies may generate distinct AD phenotypes having different resistance to pharmacological treatments (Stöhr et al., 2014; Catania et al., 2015; Qiang et al., 2017; Rasmussen et al., 2017; Di Fede et al., 2018). Since different mAbs are directed against different conformational epitopes, it is reasonable to expect a synergism from the combined use of multiple antibodies (Marciani, 2019).

Hence, it is premature to conclude from the past failures of AD immunotherapy that Aβ is the wrong therapeutic target for vaccine development. These failures, at least in part, may be due to the use of inappropriate immunogens and adjuvants, incorrect timing of intervention, and/or wrong brain delivery strategies—only approximately 0.1% of mAbs cross the blood-brain barrier (BBB; Lemere, 2013; van Dyck, 2018). Ongoing trials in AD preclinical phases to evaluate the safety, tolerability, and efficacy of mAbs in asymptomatic individuals at risk for developing AD based on PET-amyloid load, or with dominantly inherited forms of AD, will provide useful information on the validity of such strategy (Bateman et al., 2017).

### Other Therapeutic Strategies Targeting Aβ

Beyond immunotherapy, Aβ-targeting compounds that have been tested or are currently under assessment include *drugs interfering with APP processing*—i.e., α-secretase activators, β-secretase inhibitors, γ-secretase modulators—and *inhibitors of Aβ aggregation* (Citron, 2010; Suzuki et al., 2017; Umar and Hoda, 2017), based on the view that an increased amyloid production favors the onset of AD in animal models and humans carrying genetic defects in the three causative genes associated with early-onset AD, i.e., APP, presenilin 1 and 2 (Selkoe, 1991; Hardy and Selkoe, 2002; Kunkle et al., 2019).

Activation of α-secretase prevents the formation of toxic Aβ peptides and promotes the secretion of neurotrophic sAPPα by cleaving APP within the Aβ sequence (Beeg et al., 2016). A limited number of activators of α-secretase reached clinical testing and displayed fewer side effects in comparison with β- and γ-secretase inhibitors (GSIs) without showing efficacy on primary endpoints; some of them are currently under evaluation (Pinheiro and Faustino, 2019).

Inhibitors of β-secretase enzymes (BACE1 and BACE2) were proposed as disease-modifying drugs in AD following the preclinical observation that knockout mice do not develop cerebral amyloidosis (Dominguez et al., 2005). Nonetheless, most BACE inhibitors tested in controlled trials failed to prevent cognitive decline. This lack of efficacy has been attributed to their use in advanced stages of the disease (Pinheiro and Faustino, 2019). Actually, the only remaining trials with a BACE inhibitor (Elenbecestat) have been very recently halted for safety reasons^1^.

GSIs were developed to decrease Aβ production by inhibiting intramembrane APP cleavage (He et al., 2010). However, they are associated with severe gastrointestinal/immunological side effects due to abnormal processing of the Notch1 transmembrane receptor, which is also a substrate of γ-secretase, leading to increased risk of serious adverse events, including infections and skin cancers (Penninkilampi et al., 2016). These data shifted the interest of researchers to more selective GSIs (Teranishi et al., 2015) and modulators of γ-secretase (Xia, 2019) to overcome the side effects of first-generation GSIs. However, when tested in humans, even Notch-sparing GSIs failed to efficiently contrast the disease or their use was early terminated due to unacceptable side effects, especially on liver function (Doody et al., 2013; Pinheiro and Faustino, 2019).

Another anti-amyloid strategy is based on *inhibitors of Aβ aggregation*. Two main classes of molecules were developed in the last decades against AD: (i) short synthetic β-sheet breaker peptides; and (ii) nonpeptidic small drugs. However, the former generally showed poor pharmacokinetic profile, including low solubility, poor oral bioavailability and BBB permeability and elicitation of adverse immunogenic/inflammatory responses (Re et al., 2010; Hamley, 2012), and have not yet been pursued in clinical trials (Pinheiro and Faustino, 2019), with some exceptions (Shea et al., 2019). The latter, which include naturally occurring flavonoids and polyphenols, ω-3 polyunsaturated fatty acids (e.g., docosahexaenoic acid), natural organic dyes, some drugs (e.g., rifampicin and tetracycline antibiotics), ionic surfactants (e.g., sodium dodecyl sulfate and hexadecyl-*N*methylpiperidinium bromide) and several sulfur-containing compounds (e.g., α-lipoic acid and *N*-acetylcysteine) were reported to have some benefits in animal models (Ladiwala et al., 2011; Giorgetti et al., 2018) and in few clinical trials (Salloway et al., 2011; Cummings et al., 2016; Hey et al., 2018). Important limitations for the application of these group of compounds into clinical practice come from their nonspecific targets. The employment of computational approaches combined with biophysical/biochemical methods will likely help to clarify the mechanisms of interaction with targets of amyloid cascade, providing more effective drugs in the near future (Giorgetti et al., 2018).

A similar strategy based on the use of *all-D* peptides able to hinder Aβ nucleation/polymerization led recently to the design of an ongoing and promising trial (PRI-002 from PRIAVOID; Agerschou et al., 2019; Zhang T. et al., 2019).

A recent Aβ-targeting approach was proposed by our group. It is based on the use of a naturally occurring genetic variant of Aβ, consisting of an alanine-to-valine substitution at position 2 of the Aβ sequence (Aβ_A2V_). This variant—that in the homozygous state is pathogenic—is instead surprisingly protective against AD in Aβ_A2V_ heterozygous carriers (Di Fede et al., 2009; Diomede et al., 2014). The “Aβ_A2V_-based strategy” has been tested in preclinical studies showing promising results in preventing Aβ aggregation and cerebral amyloid deposition, synaptic impairment and cognitive decline (Di Fede et al., 2012, 2016; Cimini et al., 2016).

### Other Approaches Based on Amyloid Cascade Targets

Several studies in cell and animal models indicated that oligomeric, soluble Aβ is the primary driving force of AD pathogenesis but its main neurodegenerative changes are mediated at least partially by *tau protein* (Brandt and Bakota, 2017). Hence, tau and microtubules were emphasized as a target for therapeutic intervention in AD (Brandt and Bakota, 2017). The most promising tau-targeting strategies are inhibition of tau phosphorylation, proteolysis and aggregation, promotion of intracellular and extracellular tau clearance, prevention of tau spreading and stabilization of microtubules (Šimić et al., 2016). Initially, anti-tau therapies were based mainly on inhibition of kinases or tau aggregation or on stabilization of microtubules, but most of these approaches have been discontinued because of toxicity and/or lack of efficacy (Congdon and Sigurdsson, 2018). Immunotherapy against tau is now under evaluation in clinical trials as an approach potentially able to interfere with more than one of the above-cited mechanistic events (Hoskin et al., 2019). However, current tau-based immunotherapy programs are raising additional questions concerning the choice of the most efficient epitopes, the induction of extracellular vs. intracellular clearance of tau protein, the strength of the affinity of mAbs for tau, which can deeply modify the efficacy and safety of tau-targeting strategy (Golde, 2014; Pedersen and Sigurdsson, 2015; Sigurdsson, 2016). An alternative promising approach is the use of antisense oligonucleotides to decrease the expression of tau. This strategy is currently under evaluation in clinical trials for AD (DeVos et al., 2017).

### Alternative Pathogenic Hypotheses and Therapeutic Targets

AD patients have lower levels of ACh and an impaired cholinergic transmission, resulting in learning and memory dysfunction. The possibility of modulation of these related events gave rise to the “*Cholinergic Hypothesis*” for AD, which calls for enhancing cholinergic neurotransmission by the inhibition of the enzyme responsible for the metabolic breakdown of ACh (de Freitas Silva et al., 2018). Drugs currently used in the treatment of AD are mainly based on this evidence. They are acetylcholinesterase inhibitors: donepezil, rivastigmine, galantamine (just to enhance cholinergic neurotransmission), and the NMDAR antagonist memantine (addressing dysfunctional glutamatergic neurotransmission). The latter found its rationale on evidence that most neurotoxic events in AD are mediated by glutamate receptors (Zhang et al., 2016). However, these drugs do not prevent or reverse the progression of the disease, providing only symptomatic relief and/or short-term benefits (Pinheiro and Faustino, 2019).

*Neuroinflammation* is a common feature of AD and other neurodegenerative diseases, and increasing evidence indicates that neuroinflammatory processes contribute to and modulate disease pathogenesis (Walter et al., 2017; Heneka et al., 2014). However, thus far, most experimental single-target drugs that are effective in animal models were not effective in AD clinical trials (Van der Schyf, 2011). This failure was ascribed to problems in reaching or binding to the human targets *in vivo*, delayed timing of intervention, inappropriate design of clinical trial or lack of strength of the drug-target interaction to reduce the signs and symptoms of the disease (Wenzel and Klegeris, 2018). Another complication of the anti-inflammatory approach comes from the unclear role of microglia and the double-edged sword of its interplay with Aβ since both detrimental and safe effects are attributed to microglial cells in AD brains (Hansen et al., 2018; McQuade and Blurton-Jones, 2019). However, a multi-target approach against players of neuroinflammation—such as Cathepsin B, mitogen-activated protein kinases (MAPKs), monoacylglycerol lipase—was proposed as a disease-modifying therapy for AD and could theoretically overcome the limitations of the neuroinflammatory single target strategies (Jalili-Baleh et al., 2018; Wenzel and Klegeris, 2018).

The involvement of “*biometals*” in AD pathogenesis was indicated by studies showing that metal ions, namely Cu^2+^, Zn^2+^ and Fe^3+^, are known to promote the oligomerization of monomeric Aβ and to induce oxidative stress in AD brain (Grasso et al., 2012). So, metal chelation was employed as a strategy for the development of AD therapeutics based on its ability to reverse metal-induced Aβ aggregation in human brain tissue (Ayton et al., 2015). The potential for metal-based drug therapy is likely not yet fully explored by clinical trials, despite some encouraging results in humans showing mitigation of cognitive dysfunctions in AD patients (Faux et al., 2010).

It is well recognized that *oxidative stress* can contribute to aging-related neurodegenerative diseases including AD. It is also well accepted that natural compounds and minerals are powerful antioxidants that offer health benefits against several different degenerative disorders, hence the increasing interest in developing anti-oxidative therapeutics for AD. Unfortunately, there are still contradictory and inconsistent reports on the possible benefits of anti-oxidative supplements (Poprac et al., 2017; Thapa and Carroll, 2017).

## Multi-Target Approaches for AD

Despite extensive efforts and investments, the AD scientific community has been frustrated by the lack of clinical outcomes and research breakthroughs that have been translatable to clinical application. However, important suggestions come from the critical analysis of such flop. Several features contributed to the failure of experimental drugs against AD, including inadequate preclinical data, poor BBB penetration, low therapeutic window, inadequate patient selection and/or inaccurate diagnosis (Doig et al., 2017; Pinheiro and Faustino, 2019). The timing of an Aβ- or tau-targeted intervention has proven critical for clinical response since once Aβ-induced synaptic dysfunction and extensive neurodegeneration occur, they can no longer be reversed by simply reducing brain amyloid burden (Cao et al., 2018). This paradigm has shifted clinical trials from late clinical AD dementia to the early, asymptomatic stages of the disease (Pinheiro and Faustino, 2019). However, this time shift is likely still not enough.

**Figure 1:**
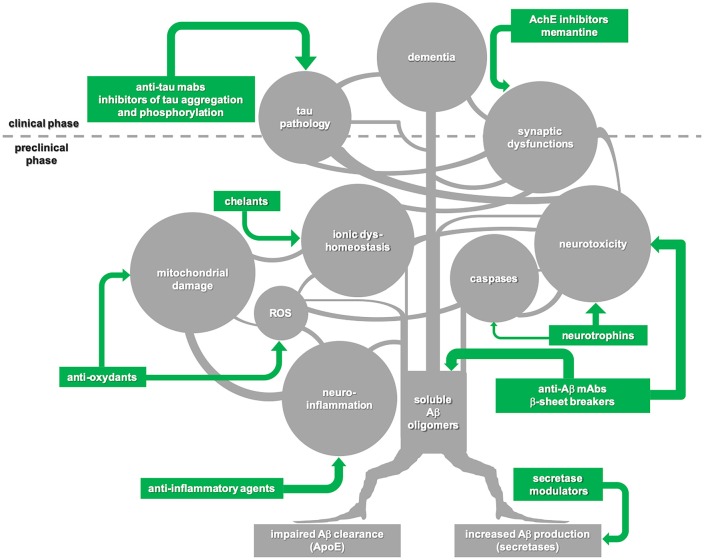
Schematic illustration of the “amyloid cascade hypothesis” that is viewed here as a tricky multi-branched process where different molecular events evolve autonomously, once triggered by the initial generation of soluble amyloid-β (Aβ) oligomers. Tau-mediated neuronal damage and neuronal toxicity induced by activation of caspases and neuroinflammation, oxidative stress with mitochondrial dysfunction, dyshomeostasis of biometals and synaptic failure are self-dependent players which synergistically sustain the onset and progression of the disease. Following this view, disease-modifying treatments for Alzheimer’s disease (AD) should be based on combined approaches against multiple targets to be successful. Moreover, taking into account that the majority of the key events in the cascade begin during the preclinical phase of the disease, a preventive multi-target strategy promises to be much more effective in comparison with late, single-target approaches.

In our opinion, amyloid cascade should be interpreted not as a linear chain of consequent steps but rather as a multi-branched network of events triggered by the formation of soluble Aβ oligomers and each characterized by self-progression (Figure 1). In this view, to substantially interfere with the molecular machinery initiated by Aβ, it is far not enough to stop the amyloid pathology—unless this is achieved very early, in the preclinical phase of the disease. More chances of being successful may come from combined approaches targeting multiple molecular players of the amyloid cascade.

Current drug design strategies are based on “*one drug-one target”* paradigm (Schneider et al., 2014), that until now failed to provide effective treatments against AD, due to the multifactorial nature of the disease (Kumar et al., 2018; Ibrahim and Gabr, 2019). Multi-target approaches for the rational design of novel drug candidates, also called multitarget-directed ligands (MTDL) strategies, have been used to develop a variety of hybrid compounds capable to act simultaneously in diverse biological targets (Viegas-Junior et al., 2007; Hughes et al., 2016; Agatonovic-Kustrin et al., 2018; Batool et al., 2018; de Freitas Silva et al., 2018; González et al., 2019). They are mostly driven by computational drug designing methods capable of assisting drug discovery (Anastasio, 2015). However, complementary tools and expertise are requested to optimize multi-target drug development (Kumar et al., 2018), including more appropriate animal models, which can fully recapitulate the molecular events of amyloid cascade (Moreno-Gonzalez and Soto, 2012; Drummond and Wisniewski, 2017).

The design of MTDLs is at the beginning of its history even if, in the recent years, several compounds (Viegas et al., 2005; Guzior et al., 2015; Romero and Marco-Contelles, 2017; Umar and Hoda, 2017; Zhang P. et al., 2019) retaining at the same time anti-aggregation properties against Aβ and cholinesterase inhibition activity or coupling modulation of serotonin and ACh pathways with the release of soluble forms of APP (sAPPα)—having neurotrophic properties—or combining chemical structures able to interact with monoamine oxidase and amyloid-binding alcohol dehydrogenase (Hroch et al., 2017) have been developed and tested in preclinical studies showing promising results (Lecoutey et al., 2014; Rochais et al., 2015; Hatat et al., 2019). However, until now no multi-target compounds have been successfully translated to the clinical context.

## Concluding Remarks

In conclusion, the multifactorial nature of AD pathophysiology supports the design of druggable compounds which can be translated into new effective and well-tolerated drugs able to interact synergistically with different targets, modulating different interconnected molecular pathways related to the disease onset and progression. There is increasing evidence in the literature in favor of the beneficial role of multi-target strategy in the cure of multifactorial diseases. In this context, several substances with multi-target activity were discovered and several of them showed interesting pharmacological profiles, making them possible drug candidates (de Freitas Silva et al., 2018), even if their development can be slowed down by the fact that MTDLs may require different concentrations to tackle different pathways. However, this disadvantage can be overcome by the combined use of multiple drugs rather than a single multi-target drug. To our knowledge, combined multi-target disease-modifying treatments against the most relevant molecular players of AD have not yet tested in controlled clinical trials. It is time for a coordinated multi-target attack on amyloid-β cascade in AD.

## Author Contributions

MC wrote the manuscript and designed the figure. GF revised and edited the manuscript. GG, MS and FT supervised the work.

## Conflict of Interest

GF and FT have an issued (0001383392) and a pending patent (102019000010722), both related to this work. MS has a pending patent (102019000010722) related to this work. The remaining authors declare that the research was conducted in the absence of any commercial or financial relationships that could be construed as a potential conflict of interest.

## Footnotes

^1^https://www.alzforum.org/news/research-news/end-bace-inhibitors-elenbecestat-trials-halted-amid-safety-concerns
